# Severe COVID-19 Infection Management in a Patient with Mild Haemophilia—A Case Report

**DOI:** 10.3390/hematolrep14020015

**Published:** 2022-03-30

**Authors:** Saša Anžej Doma, Milica Lukič

**Affiliations:** 1 Hematology Department, UMC Ljubljana, 1000 Ljubljana, Slovenia; 2 Faculty of Medicine, University of Ljubljana, 1000 Ljubljana, Slovenia; milica.lukic@kclj.si; 3 Infectious Diseases Department, UMC Ljubljana, 1000 Ljubljana, Slovenia

**Keywords:** haemophilia, SARS-CoV-2, thromboprophylaxis

## Abstract

Patients with haemophilia present a significant challenge when admitted into the intensive care unit. To prevent haemorrhagic complications related to the infection or due to invasive procedures factor (F) VIII/IX must be substituted. As thromboembolic complications are frequent among critically ill COVID-19 patients, thromboprophylaxis is also applied to patients with haemophilia. This requires careful monitoring of FVIII/IX activity as well as other haemostatic parameters, such as D-dimer and antiXa. We describe a 44-year old patient with mild haemophilia A (FVIII activity of 6%), who required a prolonged intensive care unit stay due to a severe SARS-CoV-2 infection. FVIII was substituted via boluses, and dalteparin was given according to recommendations. The patient successfully recovered from the disease.

## 1. Introduction

Patients with haemophilia (PWHs) are exposed to SARS-CoV-2 infection (COVID-19) similarly to the general population [1]. As critically ill Covid-19 patients with respiratory failure frequently suffer from thrombotic events [2,3], thromboprophylaxis is also applied to PWHs, with a sufficient factor coverage [4]. In contrast to cardiovascular risk factors, such as hypertension, obesity, diabetes, etc., haemophilia by itself is not a risk factor for a severe COVID-19 disease [1,4,5]. However, the management of PWHs admitted to the intensive care unit (ICU) can be complicated by the need to monitor factor (F) VIII or FIX levels and the appropriate substitution. Despite the published general recommendations on the management of PWHs and acute COVID-19 infection [4,6], there is a paucity of clinical reports with regards to this clinical scenario [7,8,9].

We describe a critically ill COVID-19 patient with a mild haemophilia A, morbid obesity and acute respiratory distress syndrome (ARDS) who required prolonged FVIII substitution due to intensive treatment of SARS-CoV-2 infection and who successfully recovered from the disease. Octocog alfa was administered by bolus injections, and dalteparin was applied accordingly [4].

## 2. Case Description

A 44-year old patient with mild haemophilia A (FVIII activity 6%, missense mutation in exon 26, c.6977G>A, p.Arg2326Gln) with arterial hypertension, obstructive sleep apnoea and morbid obesity (body weight 130 kg, body mass index 45 kg/m^2^), previously treated episodically with octocog alfa, was admitted to the emergency department due to severe respiratory failure. He had an eight-day history of fever, malaise and cough, and SARS-CoV-2 infection was confirmed by a positive reverse transcription polymerase chain reaction (RT-PCR) test. On admission, he had an oxygen saturation of 73%, while receiving oxygen 15 L/min via a non-rebreather mask. The laboratory parameters on admission showed a white blood cell count of 7.1 × 10^9^/L (4.0–10.0 × 10^9^/L), lymphopenia 0.5 × 10^9^/L (1.1–3.5 × 10^9^/L), C-reactive protein of 245 mg/L (<5), procalcitonin of 0.22 µg/L (<0.24), lactate dehydrogenase of 16.16 µkat/L (<4.13), creatin kinase of 22.72 µkat/L (<2.85) and troponin of 187 ng/L (<58). The haemoglobin level and platelet count were normal (157 g/L and 188 × 10^9^/L, respectively). D-dimer and fibrinogen levels were elevated (1.268 mg/L (<0.242) and 6.4 g/L (1.8–3.5), respectively), the prothrombin time was normal (1.13 (0.70–1.30), and the activated partial prothrombin time (APTT) was prolonged (47 s (25.9–36.6) but did not differ from the usual patient’s baseline value. The patient was immediately transferred to ICU and intubated after administration of 4000 IU of octocog alfa. In line with local guidelines, antiviral therapy with remdesivir 200 mg, followed by 100 mg daily and anti-inflammatory therapy with methylprednisolone 1 mg per kg per day was initiated, both intravenously. The chest X-ray showed bilateral alveolar infiltrates. Due to a possible concomitant bacterial pneumonia, amoxicillin/clavulanate was added. The patient was ventilated according to standards of lung protective ventilation, and ARDS was classified as severe (PaO2/FiO2 97 mmHg at peak end expiratory pressure of 18 cmH_2_O). 

During the ICU stay, octocog alfa was administered intravenously by bolus doses every 12 h with a target FVIII activity between 50–100%. FVIII was measured by a one-stage APTT-based assay. The inhibitors to FVIII were also tested and were negative. 

**Figure 1:**
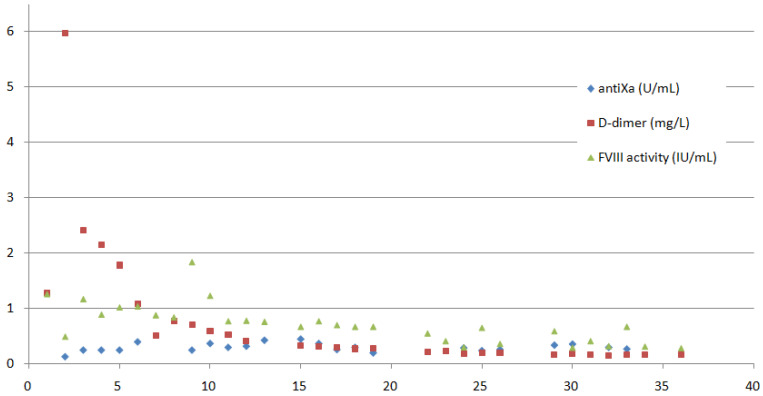
FVIII activity (IU/mL), D-dimer (mg/L) and antiXa (U/mL) measurements on the *y*-axis are presented throughout the 36-day long hospitalisation (the *x*-axis demonstrates the time in days). The patient was mechanically ventilated until day 18 of the hospitalisation; FVIII and dalteparin were dosed twice daily until day 22, and from then on once daily.

Antithrombotic prophylaxis with dalteparin 5000 IU was applied twice daily (1–4 h after the administration of actocog alfa). FVIII activity, d-dimer and antiXa were measured daily; the values are presented in Figure 1. FVIII was in the desired range most of the time (50–100%). FVIII activity was usually measured at near-trough levels, sometimes also 30 min after the administered dose to assess peak levels, which did not exceed 120%. On the second day, D-dimer levels increased to up to 35 times the upper normal level. The compression and duplex ultrasound examination of proximal veins excluded proximal deep vein thrombosis, and the computed tomography pulmonary angiography excluded pulmonary embolism. During the ICU stay, D-dimer values gradually decreased to 0.266 mg/L. Fibrinogen decreased to normal levels in a few days after admission but increased again after two weeks (to a maximum of 5.56 g/L).The platelet count and prothrombin time remained in a normal range. The renal function was normal. 

On day 9, a complication regarding the octocog alfa application occurred, and the patient received 5000 IU instead of 500 IU. This resulted in a rise of FVIII activity to 184%. A higher dose of dalteparin (7500 IU) was given an hour afterwards, and FVIII was stopped for one day. The patient was monitored for possible signs of clot formation, but the clinical course was uneventful. Moreover, the values of D-dimer remained stable, and no other haemostatic disturbances occurred due to this event. 

The patient was successfully weaned from mechanical ventilation after sixteen days. Subsequently, he was transferred to a hospital ward where he remained for an additional three weeks, being slowly weaned from oxygen and rehabilitating. During that time, octocog alfa was administered once daily to maintain FVIII levels above 30%. Dalteparin 7500 IU was applied once daily, 30 min following octocog alfa, until discharge from the hospital on day 36. The patient experienced no bleeding events during the whole hospitalisation and no thrombotic events after discharge.

## 3. Discussion

COVID-19-associated coagulopathy recently emerged as a complication of severe COVID-19, presenting with pulmonary microvascular thromboses and systemic thromboembolic manifestations. It is a consequence of the interplay between inflammation and coagulation, characterized by elevated fibrinogen, FVIII, von Willebrand factor and D-dimer. While PT, APTT and platelet count are usually not affected, increased D-dimer is the most significant change predicting thromboembolic complications [1,6]. Thromboprophylaxis, preferably low molecular weight heparin (LMWH) should be prescribed to all hospitalised patients with COVID-19 infection. In the case of a confirmed thromboembolic event, therapeutic anticoagulation is prescribed [10,11]. PWHs in the ICU present a particular challenge as they require sufficient factor VIII/FIX coverage for invasive procedures as well as for safe thromboprophylaxis [1,4,6]. For a critically ill patient with haemophilia A, trough and peak levels of 50–100% for FVIII are recommended [4,6]. This requires regular haemostasis monitoring and a good collaboration between a haemophilia-treating haematologist and ICU staff. 

The administration of FVIII or FIX by continuous infusion generally offers a more constant level of protection and usually reduces the costs of treatment [12], especially in patients with severe haemophilia, but jit requires a separate intravenous line dedicated to this drug. The bolus administration of FVIII or FIX can be preferred in the ICU environment if infusion pumps are lacking, also instantly providing desired peaks before invasive procedures. FVIII boluses are usually given over 12h intervals, so the nursing staff does not need to enter the rooms frequently. Previously investigated, continuous infusion is not considered a risk factor for inhibitor development [13]. However, for our patient, who did not have a high-risk genotype [14] and had >50 prior FVIII exposure days, we chose bolus administration mainly to avoid the risk of a possible discontinuing of FVIII infusion due to the application of many drugs that critically ill patients receive. We continued with the same FVIII product (octocog alfa) the patient used before. The administration of octocog alfa every 12 h provided good protection without peaks that were too high. In our patient, the target troughs for FVIII substitution were chosen according to the recommendations [4,6]:above 50% while the patient was mechanically ventilated, and above 30% after the transfer from the ICU to the hospital ward. On occasion, higher levels of FVIII of up to 120% were measured, which we believe was appropriate in view of the extensive injury of his lungs necessitating high inspiratory pressure ventilation as well as invasive procedures (such as arterial punctures for blood gas analysis, central venous catheter insertion), frequently performed in the ICU without a previous FVIII measurement or consultation with the haematologist. Dalteparin was prescribed as recommended [6,10,11]; intermediate prophylactic doses were chosen because of the patient’s obesity, a known risk factor for thrombosis [3,11]. Although the most appropriate LMWH dosing in patients with obesity remains unclear [3,6,15], our choice of dose escalation was based on the decision of a multidisciplinary team considering both the patient’s high D-dimer on admission as well as his risk factors for thromboembolic complications [2,3,16]. As he later experienced no bleedings, we continued with the dosing despite a decrease of D-dimer, keeping in mind that in PWHs thrombosis is much more difficult to treat than bleeding. AntiXa was measured 3 h and 5 h after the twice-daily and once-daily administration of dalteparin, respectively, with the aim of maintaining antiXa below the therapeutic range. 

A dose-related incident of a higher-than-recommended administration of octocog alpha occurred during the ICU stay. The incident was attributed to medical staff not familiar with FVIII concentrates and a stressful working environment associated with a higher pandemic-related workload. The event was managed with a higher dose of dalteparin and did not cause a rise of FVIII over 180% or any other haemostatic disturbances. However, severe SARS-CoV infection is known to increase FVIII levels significantly (above 300%) in non-haemophilia patients [16].

## 4. Conclusions

The treatment of severe COVID-19 in patients with haemophilia A should not differ from regular COVID-19 treatment guidelines, provided that FVIII is adequately substituted. Regular measurements of haemostatic parameters as well as close contact with a haemophilia-treating haematologist are important for a favourable outcome.

## Data Availability

Data and materials are available within the text.
